# The pGinger Family of Expression Plasmids

**DOI:** 10.1128/spectrum.00373-23

**Published:** 2023-05-22

**Authors:** Allison N. Pearson, Mitchell G. Thompson, Liam D. Kirkpatrick, Cindy Ho, Khanh M. Vuu, Lucas M. Waldburger, Jay D. Keasling, Patrick M. Shih

**Affiliations:** a Joint BioEnergy Institute, Emeryville, California, USA; b Biological Systems & Engineering Division, Lawrence Berkeley National Laboratory, Berkeley, California, USA; c Department of Plant and Microbial Biology, University of California, Berkeley, California, USA; d Environmental Genomics and Systems Biology Division, Lawrence Berkeley National Laboratory, Berkeley, California, USA; e Department of Bioengineering, University of California, Berkeley, California, USA; f Department of Chemical and Biomolecular Engineering, University of California, Berkeley, California, USA; g The Novo Nordisk Foundation Center for Biosustainability, Technical University of Denmark, Kongens Lyngby, Denmark; h Center for Synthetic Biochemistry, Institute for Synthetic Biology, Shenzhen Institutes for Advanced Technologies, Shenzhen, China; i Innovative Genomics Institute, University of California, Berkeley, California, USA; University of Minnesota

**Keywords:** molecular genetics, plasmid, synthetic biology

## Abstract

The pGinger suite of expression plasmids comprises 43 plasmids that will enable precise constitutive and inducible gene expression in a wide range of Gram-negative bacterial species. Constitutive vectors are composed of 16 synthetic constitutive promoters upstream of red fluorescent protein (RFP), with a broad-host-range BBR1 origin and a kanamycin resistance marker. The family also has seven inducible systems (Jungle Express, Psal/NahR, Pm/XylS, Prha/RhaS, LacO1/LacI, LacUV5/LacI, and Ptet/TetR) controlling RFP expression on BBR1/kanamycin plasmid backbones. For four of these inducible systems (Jungle Express, Psal/NahR, LacO1/LacI, and Ptet/TetR), we created variants that utilize the RK2 origin and spectinomycin or gentamicin selection. Relevant RFP expression and growth data have been collected in the model bacterium Escherichia coli as well as Pseudomonas putida. All pGinger vectors are available via the Joint BioEnergy Institute (JBEI) Public Registry.

**IMPORTANCE** Metabolic engineering and synthetic biology are predicated on the precise control of gene expression. As synthetic biology expands beyond model organisms, more tools will be required that function robustly in a wide range of bacterial hosts. The pGinger family of plasmids constitutes 43 plasmids that will enable both constitutive and inducible gene expression in a wide range of nonmodel *Proteobacteria*.

## INTRODUCTION

Precise and reliable control over gene expression is one of the most fundamental requirements of synthetic and molecular biology (1). Consequently, there has been considerable effort toward identifying myriad genetic elements that enable researchers to regulate the strength and timing of transcription across all domains of life (2–4). The results of these efforts are often consolidated families of plasmid vectors that facilitate advanced genetic engineering, such as the BglBrick family of plasmids for Escherichia coli (5, 6) and the jStack vectors used in multiple plant species (7). However, as the field of synthetic biology moves beyond traditional model organisms, families of expression vectors must be tailored to meet the specific requirements of particular hosts. Advances in nonmodel organisms often come in the form of species- or genus-specific toolkits (8–10), though more recently, comprehensive plasmid toolkits have been developed and validated for a wide range of Gram-negative organisms (11). Resources such as the Standard European Vector Architecture (SEVA) platform provide repositories of standardized sequences and constructs (12, 13). Still, given that many bacteria require very particular combinations of promoters, origins, and selectable markers to enable controlled gene expression, there remains a need for vectors that will allow rapid prototyping of genetic circuits in understudied bacteria.

To facilitate the exploration of nonmodel hosts, we have developed a small suite of plasmids that permit both constitutive and inducible expression from the broad-host-range origin of replication BBR1 using a kanamycin selection marker. For a subset of the inducible systems that are known to work across multiple hosts, we have assembled combinatorial variants that utilize the compatible broad-host-range origin RK2 (14) as well as both spectinomycin and gentamicin selection markers. This family of plasmids, which we have named the pGinger suite, requires no assembly of these parts, can be easily cloned into via standard Gibson assembly techniques, and has both digital sequences and physical samples that can be publicly accessed through the Joint BioEnergy Institute (JBEI) registry (15).

## RESULTS

### Design and architecture of pGinger plasmids.

**FIG 1:**
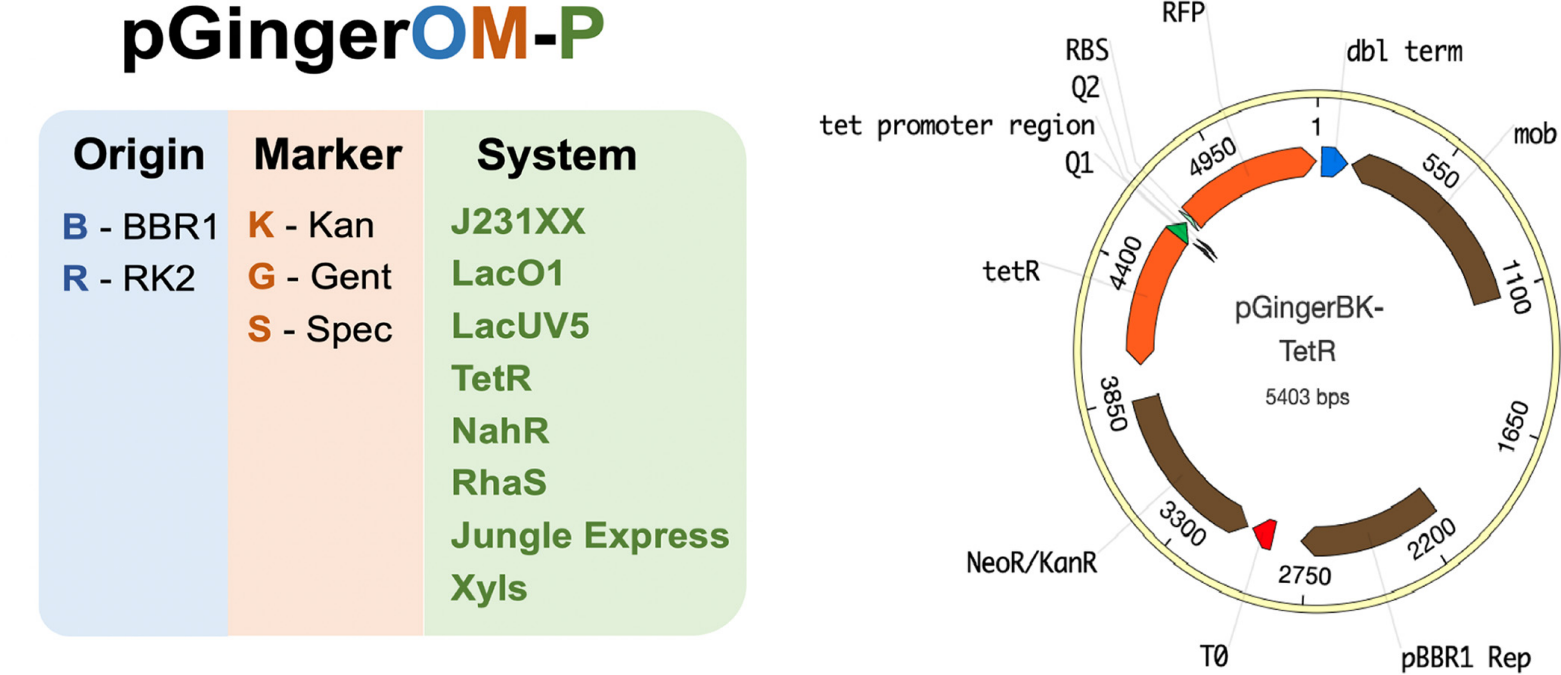
Plasmid architecture of the pGinger suite. The pGinger plasmids share a naming convention in which the first two letters after “pGinger” correspond to the origin and resistance marker, respectively, followed by the expression system. All plasmids share the same architecture as in the map of pGingerBK-TetR, whereby a conserved RBS-RFP is downstream of the promoter, followed by a strong terminator. All selectable markers are upstream of the promoter, with the origin between the marker and the RFP cassette.

All pGinger vectors express red fluorescent protein (RFP) with a consensus ribosomal binding site (RBS; TTTAAGAAGGAGATATACAT) derived from the BglBrick plasmid library. The overall conserved plasmid architecture and naming convention of the pGinger suite are shown in Fig. 1.

The BBR1 origin and kanamycin resistance cassette of relevant pGinger vectors were both derived from plasmid pBADTrfp (16). To develop a family of constitutive expression plasmids, the AraC coding sequence and promoter of pBADTrfp were replaced with 16 different synthetic promoters from the Anderson Promoter Library (http://parts.igem.org/Promoters/Catalog/Anderson). For the inducible vectors, the AraC coding sequence and promoter of pBADTrfp were replaced with the following seven inducible systems: Jungle Express, derived from pTR_sJExD-rfp (17); Psal/NahR, derived from pPS43 (18); Prha/RhaS, derived from pCV203 (18); Ptet/TetR, derived from pBbE2a-RFP (6); Pm/XylS, derived from pPS66 (18); LacO1/LacI, derived from pBbE6a-RFP (6); and LacUV5/LacI, derived from pBbE5a-RFP (6). Three of these inducible systems, Pm/XylS, Psal/NahR, and Prha/RhaS, utilize an activator or bifunctional transcription factor; the other systems feature transcriptional repressors. These BBR1 vectors contain the *mob* element that facilitates conjugal transfer. For four of the inducible systems (Jungle Express, Psal/NahR, LacO1/LacI, and Ptet/TetR), additional vectors were constructed that varied both the origin and antibiotic marker. All RK2 origins were derived from pBb(RK2)1k-GFPuv (8), while the gentamicin resistance cassette was derived from pMQ30 (19) and the spectinomycin resistance cassette was derived from pSR43.6 (20). The RK2 vectors do not contain the *mob* element. A full description of each pGinger vector can be found in Table 1.

**TABLE 1:** Plasmids in the pGinger suite^*a*^

Name	Origin	Marker	Promoter class	System	Inducer	JBEI ICE no.
pGingerBK-J23100	BBR1	Kanamycin	Constitutive	J23100	NA	JPUB_020797
pGingerBK-J23101	BBR1	Kanamycin	Constitutive	J23101	NA	JPUB_020799
pGingerBK-J23102	BBR1	Kanamycin	Constitutive	J23102	NA	JPUB_020815
pGingerBK-J23103	BBR1	Kanamycin	Constitutive	J23103	NA	JPUB_020801
pGingerBK-J23104	BBR1	Kanamycin	Constitutive	J23104	NA	JPUB_020803
pGingerBK-J23105	BBR1	Kanamycin	Constitutive	J23105	NA	JPUB_020817
pGingerBK-J23106	BBR1	Kanamycin	Constitutive	J23106	NA	JPUB_020793
pGingerBK-J23107	BBR1	Kanamycin	Constitutive	J23107	NA	JPUB_020819
pGingerBK-J23108	BBR1	Kanamycin	Constitutive	J23108	NA	JPUB_020821
pGingerBK-J23110	BBR1	Kanamycin	Constitutive	J23110	NA	JPUB_020805
pGingerBK-J23111	BBR1	Kanamycin	Constitutive	J23111	NA	JPUB_020807
pGingerBK-J23113	BBR1	Kanamycin	Constitutive	J23113	NA	JPUB_020809
pGingerBK-J23114	BBR1	Kanamycin	Constitutive	J13114	NA	JPUB_020811
pGingerBK-J23117	BBR1	Kanamycin	Constitutive	J13117	NA	JPUB_020795
pGingerBK-J23118	BBR1	Kanamycin	Constitutive	J23118	NA	JPUB_020823
pGingerBK-J23119	BBR1	Kanamycin	Constitutive	J23119	NA	JPUB_020813
pGingerBK-JE	BBR1	Kanamycin	Inducible	Jungle Express	Crystal violet	JPUB_020825
pGingerBK-NahR	BBR1	Kanamycin	Inducible	Psal/NahR	Salicylic acid	JPUB_020831
pGingerBK-RhaS	BBR1	Kanamycin	Inducible	Prha/RhaS	Rhamnose	JPUB_020829
pGingerBK-TetR	BBR1	Kanamycin	Inducible	Ptet/TetR	Oxytetracycline	JPUB_020835
pGingerBK-XylS	BBR1	Kanamycin	Inducible	Pm/XylS	Benzoate	JPUB_020827
pGingerBK-Lac	BBR1	Kanamycin	Inducible	LacO1/LacI	IPTG	JPUB_020833
pGingerBK-LacUV5	BBR1	Kanamycin	Inducible	LacUV5/LacI	IPTG	JPUB_020837
pGingerBG-JE	BBR1	Gentamicin	Inducible	Jungle Express	Crystal violet	JPUB_020847
pGingerBS-JE	BBR1	Spectinomycin	Inducible	Jungle Express	Crystal violet	JPUB_020855
pGingerRK-JE	RK2	Kanamycin	Inducible	Jungle Express	Crystal violet	JPUB_020871
pGingerRG-JE	RK2	Gentamicin	Inducible	Jungle Express	Crystal violet	JPUB_020881
pGingerRS-JE	RK2	Spectinomycin	Inducible	Jungle Express	Crystal violet	JPUB_020863
pGingerBG-NahR	BBR1	Gentamicin	Inducible	Psal/NahR	Salicylic acid	JPUB_020845
pGingerBS-NahR	BBR1	Spectinomycin	Inducible	Psal/NahR	Salicylic acid	JPUB_020853
pGingerRK-NahR	RK2	Kanamycin	Inducible	Psal/NahR	Salicylic acid	JPUB_020869
pGingerRG-NahR	RK2	Gentamicin	Inducible	Psal/NahR	Salicylic acid	JPUB_020879
pGingerRS-NahR	RK2	Spectinomycin	Inducible	Psal/NahR	Salicylic acid	JPUB_020859
pGingerBG-TetR	BBR1	Gentamicin	Inducible	Ptet/TetR	Oxytetracycline	JPUB_020843
pGingerBS-TetR	BBR1	Spectinomycin	Inducible	Ptet/TetR	Oxytetracycline	JPUB_020851
pGingerRK-TetR	RK2	Kanamycin	Inducible	Ptet/TetR	Oxytetracycline	JPUB_020865
pGingerRG-TetR	RK2	Gentamicin	Inducible	Ptet/TetR	Oxytetracycline	JPUB_020877
pGingerRS-TetR	RK2	Spectinomycin	Inducible	Ptet/TetR	Oxytetracycline	JPUB_020861
pGingerBG-LacO1	BBR1	Gentamicin	Inducible	LacO1/LacI	IPTG	JPUB_020841
pGingerBS-Lac	BBR1	Spectinomycin	Inducible	LacO1/LacI	IPTG	JPUB_020849
pGingerRK-LacO1	RK2	Kanamycin	Inducible	LacO1/LacI	IPTG	JPUB_020867
pGingerRG-LacO1	RK2	Gentamicin	Inducible	LacO1/LacI	IPTG	JPUB_020875
pGingerRS-LacO1	RK2	Spectinomycin	Inducible	LacO1/LacI	IPTG	JPUB_020857

a Relevant characteristics of pGinger plasmids, including origin of replication, antibiotic selection, promoter characteristics, and (if applicable) inducing molecule. JBEI public registry numbers are also included for digital accessibility. ICE, inventory of composable elements; NA, not applicable; IPTG, isopropyl-β-d-thiogalactopyranoside.

### Evaluation of constitutive expression pGinger plasmids.

**FIG 2:**
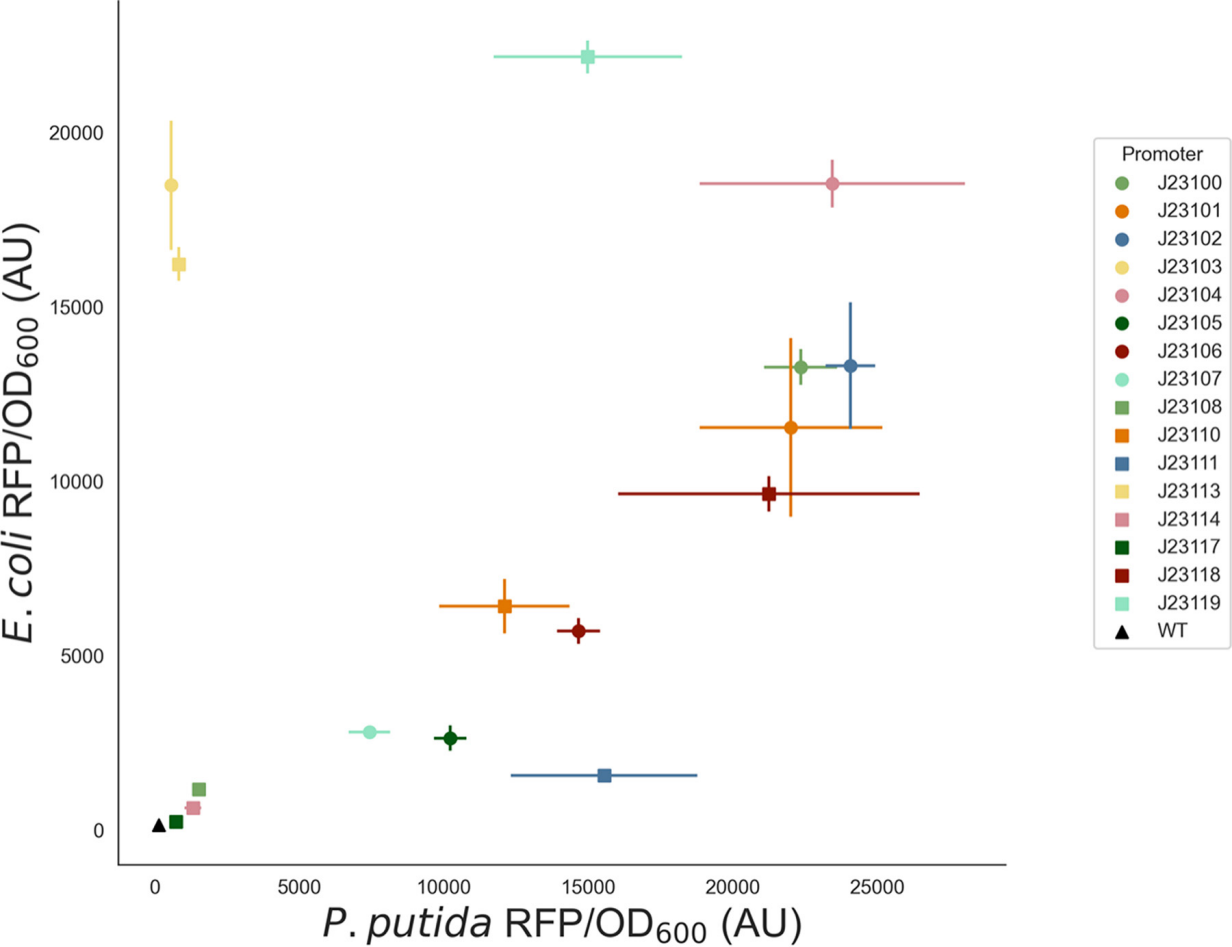
Activity of constitutive promoters in E. coli and P. putida. RFP expression normalized to cell density from Anderson promoters within either E. coli (*y* axis) or P. putida (*x* axis) is shown with standard deviations (*n* = 3). The background fluorescence of the two bacteria is indicated by “WT” (wild type). Optical density measurements are shown in Fig. S1 in the supplemental material. AU, arbitrary units.

To evaluate the relative strength of constitutive Anderson promoters in the context of the pGinger vectors, plasmids were introduced into both Pseudomonas putida and E. coli. Fluorescence was measured after growth in lysogeny broth (LB) medium for 24 h. When fluorescence was normalized to cell density, expression from Anderson promoters showed significant correlation (Spearman’s ρ = 0.49; *P = *0.045) between P. putida and E. coli (Fig. 2). Promoters J23103 and J23113 were significantly stronger in E. coli than in P. putida, while promoter J23111 was significantly stronger in P. putida. Promoter sequences and mean expression values in both E. coli and P. putida are listed in Table 2.

**TABLE 2:** Expression of pGinger Anderson promoters^*a*^

Promoter	Promoter sequence	E. coli expression	P. putida expression
J23100	TTGACGGCTAGCTCAGTCCTAGGTACAGTGCTAGC	13,267 (±517)	22,343 (±1,262)
J23101	TTTACAGCTAGCTCAGTCCTAGGTATTATGCTAGC	11,530 (±2,565)	22,010 (±3,162)
J23102	TTGACAGCTAGCTCAGTCCTAGGTACTGTGCTAGC	13,300 (±1,815)	24,067 (±858)
J23103	CTGATAGCTAGCTCAGTCCTAGGGATTATGCTAGC	18,476 (±1,857)	565 (±135)
J23104	TTGACAGCTAGCTCAGTCCTAGGTATTGTGCTAGC	18,522 (±682)	23,440 (±4,588)
J23105	TTTACGGCTAGCTCAGTCCTAGGTACTATGCTAGC	2,622 (±363)	10,220 (±558)
J23106	TTTACGGCTAGCTCAGTCCTAGGTATAGTGCTAGC	5,697 (±369)	14,659 (±748)
J23107	TTTACGGCTAGCTCAGCCCTAGGTATTATGCTAGC	2,798 (±44)	7,429 (±716)
J23108	CTGACAGCTAGCTCAGTCCTAGGTATAATGCTAGC	1,149 (±84)	1,523 (±84)
J23110	TTTACGGCTAGCTCAGTCCTAGGTACAATGCTAGC	6,402 (±782)	12,098 (±2,251)
J23111	TTGACGGCTAGCTCAGTCCTAGGTATAGTGCTAGC	1,547 (±106)	15,548 (±3,229)
J23113	CTGATGGCTAGCTCAGTCCTAGGGATTATGCTAGC	16,220 (±480)	826 (±92)
J23114	TTTATGGCTAGCTCAGTCCTAGGTACAATGCTAGC	625 (±48)	1,325 (±289)
J23117	TTGACAGCTAGCTCAGTCCTAGGGATTGTGCTAGC	229 (±25)	730 (±28)
J23118	TTGACGGCTAGCTCAGTCCTAGGTATTGTGCTAGC	9,628 (±507)	21,237 (±5,215)
J23119	TTGACAGCTAGCTCAGTCCTAGGTATAATGCTAGC	22,157 (±473)	14,979 (±3,262)
WT	NA	124 (±6)	141 (±17)

a For each Anderson promoter, the sequence is provided as well as the mean cell density-normalized RFP fluorescence in both E. coli and P. putida. Standard deviations are provided in parentheses (*n* = 3). The background fluorescence of E. coli is indicated by “WT” (wild type).

### Evaluation of inducible pGinger plasmids.

**FIG 3:**
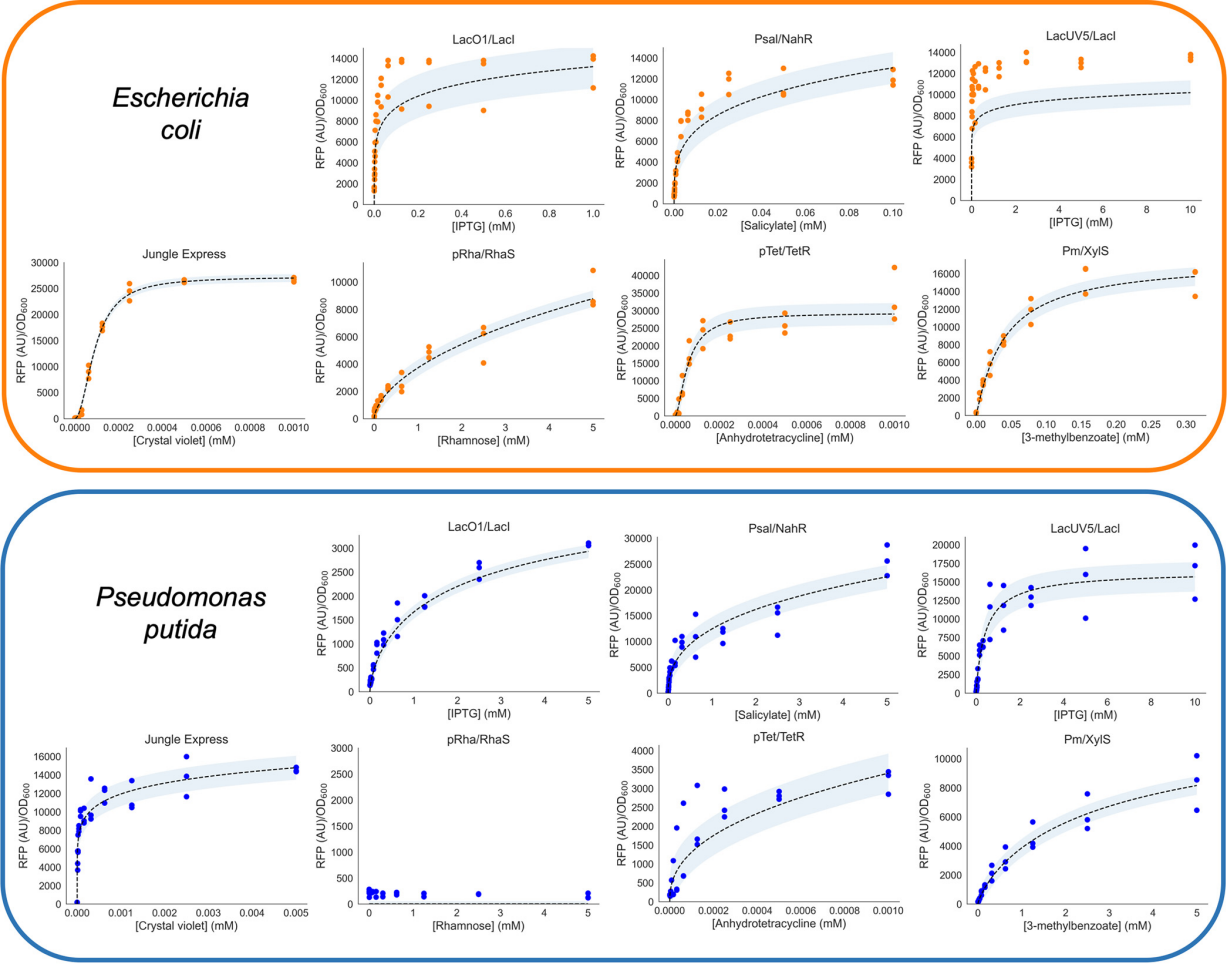
Activity of inducible systems in E. coli and P. putida. RFP expression normalized to cell density (*y* axis) from inducible systems within either E. coli (top panel in orange) or P. putida (bottom panel in blue) is shown as a function of millimolar concentration of inducer (*x* axis). Fits to the Hill equation are shown as dashed lines and shaded to show confidence intervals. Raw data points are overlaid (*n* = 3). Corresponding optical density measurements are shown in Fig. S2.

The expression of the seven inducible systems within the pGinger suite was evaluated using the BBR1 origin and kanamycin marker (pGingerBK) against a titration of the inducer in both E. coli and P. putida (Fig. 3). All systems showed inducibility in E. coli, and all but the rhamnose-inducible system Prha/RhaS showed inducibility in P. putida. Relevant expression characteristics of the inducible pGingerBK vectors in both tested bacteria are listed in Table 3. The strongest normalized expression from an inducible system in E. coli was the Ptet/TetR system, while both the strongest in P. putida were found to be Psal/NahR and Jungle Express inducible systems, which showed nearly identical maximal expression. In both bacteria, the Jungle Express system demonstrated the greatest level of induction relative to background expression.

**TABLE 3:** Inducible systems in E. coli and P. putida^*a*^

System	Organism	Background	Max expression	Max concn	Induction
LacO1/LacI	E. coli	1,510 (±186)	13,127 (±1,693)	1 mM	9×
	P. putida	137 (±5)	3,074 (±30)	5 mM	22×
Psal/NahR	E. coli	762 (±130)	12,051 (±759)	100 μM	16×
	P. putida	237 (±5)	25,697 (±2,976)	5 mM	108×
LacUV5/LacI	E. coli	3,577 (±36)	10,137 (±855)	10 mM	4×
	P. putida	203 (±53)	16,622 (±3,671)	10 mM	82×
Jungle Express	E. coli	104 (±7)	26,717 (±418)	1 μM	257×
	P. putida	176 (±5)	14,552 (±145)	2.5 μM	83×
Prha/RhaS	E. coli	172 (±42)	9,251 (±1,389)	5 mM	54×
	P. putida	NA	NA	NA	NA
Ptet/TetR	E. coli	341 (±10)	33,631 (±7,692)	1 μM	98×
	P. putida	176 (±9)	3,214 (±319)	1 μM	18×
Pm/XylS	E. coli	329 (±57)	15,280 (±1,590)	313 μM	46×
	P. putida	161 (±7)	8,401 (±1,877)	5 mM	52×

a For each inducible system on a BBR1 origin with a kanamycin marker, the experimentally observed background (uninduced) fluorescence and maximal fluorescence are given for both E. coli and P. putida. Standard deviations are provided in parentheses (*n* = 3). Additionally, the inducer concentration used to achieve maximal expression and the relative induction levels are listed. Max, maximum.

**FIG 4:**
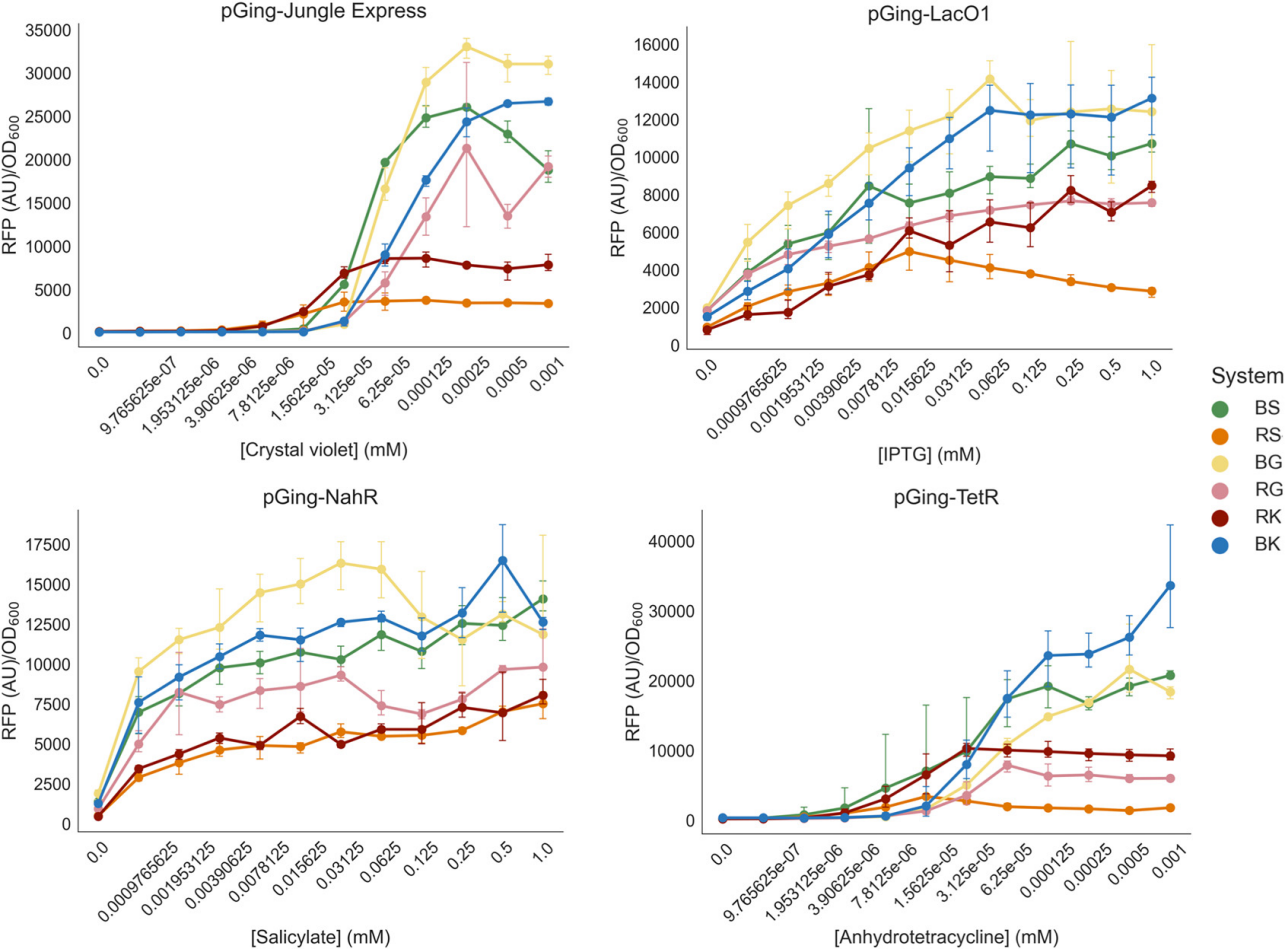
Activity of inducible pGinger variants in E. coli. For origin and selection marker pGinger variants of Jungle Express (top left), LacO1/LacI (top right), Psal/NahR (bottom left), and pTet/TetR (bottom right), dose-response curves of normalized RFP expression are shown as a function of millimolar concentration of inducer. Error bars represent standard deviations (*n* = 3). Note that the *x* axis is nonlinear. Corresponding optical density measurements are shown in Fig. S3. Results of kinetic experiments for these systems are shown in Fig. S4 to S7.

To evaluate the effects of various origin and selectable markers on expression from inducible systems, all six variants of Jungle Express, LacO1/LacI, Psal/NahR, and Ptet/TetR were investigated for their dose responses to their inducer molecules in E. coli (Fig. 4). Relevant expression parameters are listed in Table 4. In general, BBR1 variants showed greater expression than RK2 origin plasmids, which was expected given the higher copy number of BBR1 plasmids in E. coli (11). Among the pGinger Jungle Express vectors, both pGingerRS-JE and pGingerRK-JE showed dose responses distinct from those of the other vectors (Fig. 4, top left). Notably, all pGingerRS (RK2-spectinomycin) plasmids showed the lowest expression across each system tested (Table 4).

**TABLE 4:** Inducible pGinger variants in E. coli^*a*^

System	Origin	Marker	Background	Max expression	Max concn	Induction
LacO1/LacI	BBR1	Kan	1,510 (±186)	13,127 (±1,693)	1 mM	9×
LacO1/LacI	BBR1	Gent	1,976 (±70)	14,143 (±1,608)	63 μM	7×
LacO1/LacI	BBR1	Spec	1,830 (±320)	10,694 (±943)	250 μM	6×
LacO1/LacI	RK2	Kan	803 (±216)	8,213 (±721)	250 μM	10×
LacO1/LacI	RK2	Gent	1,823 (±78)	7,654 (±230)	250 μM	4×
LacO1/LacI	RK2	Spec	950 (±46)	4,967 (±895)	16 μM	5×
Psal/NahR	BBR1	Kan	1,260 (±57)	16,484 (±1,693)	500 μM	9×
Psal/NahR	BBR1	Gent	1,877 (±334)	16,317 (±1,534)	31 μM	9×
Psal/NahR	BBR1	Spec	1,372 (±84)	14,083 (±984)	1 mM	10×
Psal/NahR	RK2	Kan	445 (±39)	8,048 (±859)	1 mM	18×
Psal/NahR	RK2	Gent	934 (±56)	9,299 (±460)	1 mM	10×
Psal/NahR	RK2	Spec	482 (±69)	7,510 (±817)	1 mM	16×
Jungle Express	BBR1	Kan	104 (±6)	267,175 (±418)	1 μM	257×
Jungle Express	BBR1	Gent	136 (±19)	33,060 (±1,185)	250 nM	243×
Jungle Express	BBR1	Spec	152 (±5)	26,039 (±355)	250 nM	171×
Jungle Express	RK2	Kan	163 (±27)	8,616 (±902)	125 nM	52×
Jungle Express	RK2	Gent	138 (±51)	21,314 (±9,517)	250 nM	155×
Jungle Express	RK2	Spec	168 (±5)	3,763 (±204)	125 nM	22×
Ptet/TetR	BBR1	Kan	341 (±10)	33,631 (±7,692)	1 μM	98×
Ptet/TetR	BBR1	Gent	351 (±32)	21,646 (±5,579)	500 nM	62×
Ptet/TetR	BBR1	Spec	232 (±43)	19,224 (±3,027)	125 nM	83×
Ptet/TetR	RK2	Kan	184 (±5)	10,281 (±967)	31 nM	56×
Ptet/TetR	RK2	Gent	232 (±39)	7,883 (±865)	63 nM	34×
Ptet/TetR	RK2	Spec	197 (±5)	3,399 (±143)	16 nM	17×

a For pGinger variants of LacO1/LacI, Psal/NahR, Jungle Express, and Ptet/TetR inducible systems, the experimentally observed background (uninduced) fluorescence and maximal fluorescence in E. coli are provided. Standard deviations are provided in parentheses (*n* = 3). Additionally, the inducer concentration used to achieve maximal expression and the relative induction levels are listed. Kan, kanamycin; Gent, gentamicin; Spec, spectinomycin.

## DISCUSSION

The pGinger suite of plasmids offers researchers an array of small, preassembled vectors that will permit rapid identification of useful genetic elements in diverse Gram-negative bacteria due to the use of broad-host-range origins (RK2) and selectable markers known to work across many species (kanamycin, spectinomycin, and gentamicin). The compatibility of RK2 and BBR1 origins may also permit researchers to introduce multiple pGinger vectors into a single strain simultaneously (14). In combination with other recent plasmid suites that have been publicly released, the pGinger plasmids have the potential to facilitate more advanced synthetic biology and metabolic engineering efforts in bacterial species that have been traditionally understudied.

## MATERIALS AND METHODS

### Strains and media.

Cultures were grown in lysogeny broth (LB) Miller medium (BD Biosciences, USA) at 37°C for E. coli XL1-Blue (QB3 MacroLab, USA) and 30°C for P. putida KT2440 (ATCC 47054). The medium was supplemented with kanamycin (50 mg/L; Sigma-Aldrich, USA), gentamicin (30 mg/L; Fisher Scientific, USA), or spectinomycin (100 mg/L; Sigma-Aldrich), when indicated. All other compounds were purchased through Sigma-Aldrich.

### Plasmid design and construction.

All plasmids were designed using Device Editor and Vector Editor software, while all primers used for the construction of plasmids were designed using j5 software (15, 21, 22). Plasmids were assembled via Gibson assembly using standard protocols (23). Plasmids were routinely isolated using the Qiaprep Spin miniprep kit (Qiagen, USA), and all primers were purchased from Integrated DNA Technologies (IDT; Coralville, IA). The fluorescent protein used in all plasmids was mRFP1 (24).

### Characterization assays.

To characterize RFP expression from the vectors, we measured optical density and fluorescence after growth in 96-well plates for 24 h. First, overnight cultures were inoculated into 5 mL of LB medium from single colonies and grown at 30°C or 37°C. These cultures were then diluted 1:100 into 500 μL of LB medium with the appropriate antibiotic in 96 square v-bottom deep-well plates (Biotix DP22009CVS). For characterization of the inducible systems, inducer was added to wells in the first column of the plate at the maximum concentration tested and diluted 2-fold across the plate until the last column, which was left as the zero-inducer control. Plates were sealed with a gas-permeable microplate adhesive film (Axygen BF400S) and cultures were grown for 24 h at either 30°C or 37°C with shaking at 200 rpm. Optical density was measured at 600 nm, and fluorescence was measured at an excitation wavelength of 535 nm and an emission wavelength of 620 nm. All data were analyzed and visualized using custom Python scripts using the SciPy (25), NumPy (26), Pandas, Matplotlib, and Seaborn libraries. Fits to the Hill equation were done as previously described (27).

### Plasmid and sequence availability.

All strains and plasmid sequences from Table 1 can be found via the following link to the JBEI Public Registry: https://public-registry.jbei.org/folders/771. Users can request strains via an material transfer agreement (MTA).
