# Supplementary material for: The pGinger Family of Expression Plasmids
Source: Microbiol Spectr. 2023 May 22;11(3):e00373-23. doi: 10.1128/spectrum.00373-23 (PMC10269703; doi:10.1128/spectrum.00373-23)
Supplement: Supplemental file 1 — Fig. S1 to S7. Download spectrum.00373-23-s0001.docx, DOCX file, 3.2 MB [file spectrum.00373-23-s0001.docx]

**The pGinger family of expression plasmids: Supplemental Material**

Allison N. Pearson^1,2,3*^, Mitchell G. Thompson^1,4*^, Liam D. Kirkpatrick^1,4^, Cindy Ho^1,2^, Khanh M. Vuu^1,4^, Lucas M. Waldburger^1,2,5^, Jay D. Keasling^1,2,6,7,8✝,^, Patrick M. Shih^1,3,4,9✝^

^1^Joint BioEnergy Institute, 5885 Hollis Street, Emeryville, CA 94608, USA.

^2^Biological Systems & Engineering Division, Lawrence Berkeley National Laboratory, Berkeley, CA 94720, USA.

^3^Department of Plant and Microbial Biology, University of California, Berkeley, CA 94720, USA

^4^Environmental Genomics and Systems Biology Division, Lawrence Berkeley National Laboratory, Berkeley, California, USA

^5^Department of Bioengineering, University of California, Berkeley, California, USA

^6^Department of Chemical and Biomolecular Engineering, University of California, Berkeley, CA 94720, USA

^7^The Novo Nordisk Foundation Center for Biosustainability, Technical University of Denmark, Denmark

^8^Center for Synthetic Biochemistry, Institute for Synthetic Biology, Shenzhen Institutes for Advanced Technologies, Shenzhen, China

^9^Innovative Genomics Institute, University of California, Berkeley, CA

*Allison N. Pearson and Mitchell G. Thompson contributed equally to this manuscript

^✝^Correspondence should be addressed to either Jay D. Keasling (jdkeasling@lbl.gov) or Patrick M. Shih (pmshih@lbl.gov)

**
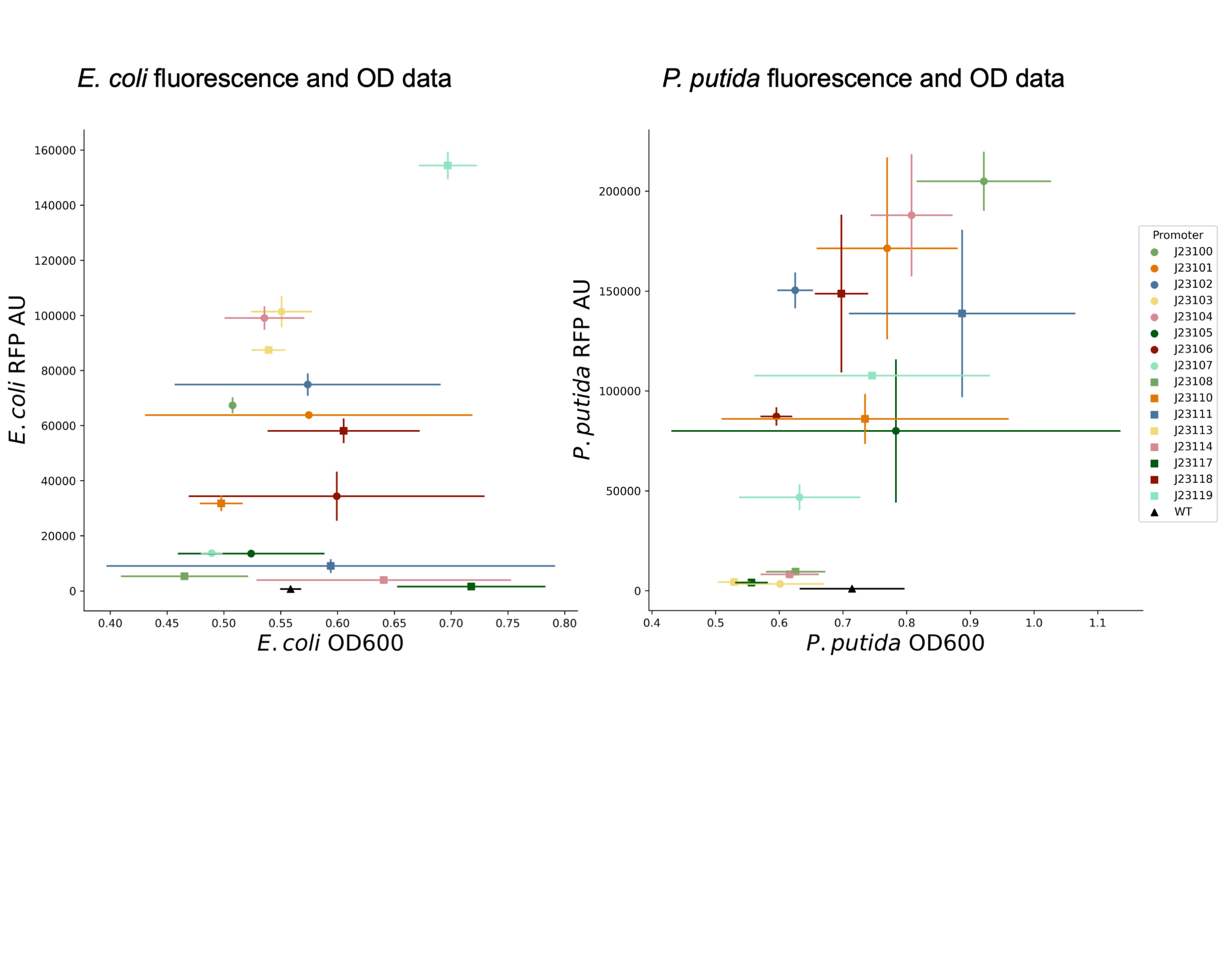
Figure S1:** Optical density and fluorescence measurements after 24 hours of growth, corresponding to the normalized fluorescence data presented in Figure 2. Error bars represent standard deviation (n=3, error bars = std. dev.)

**
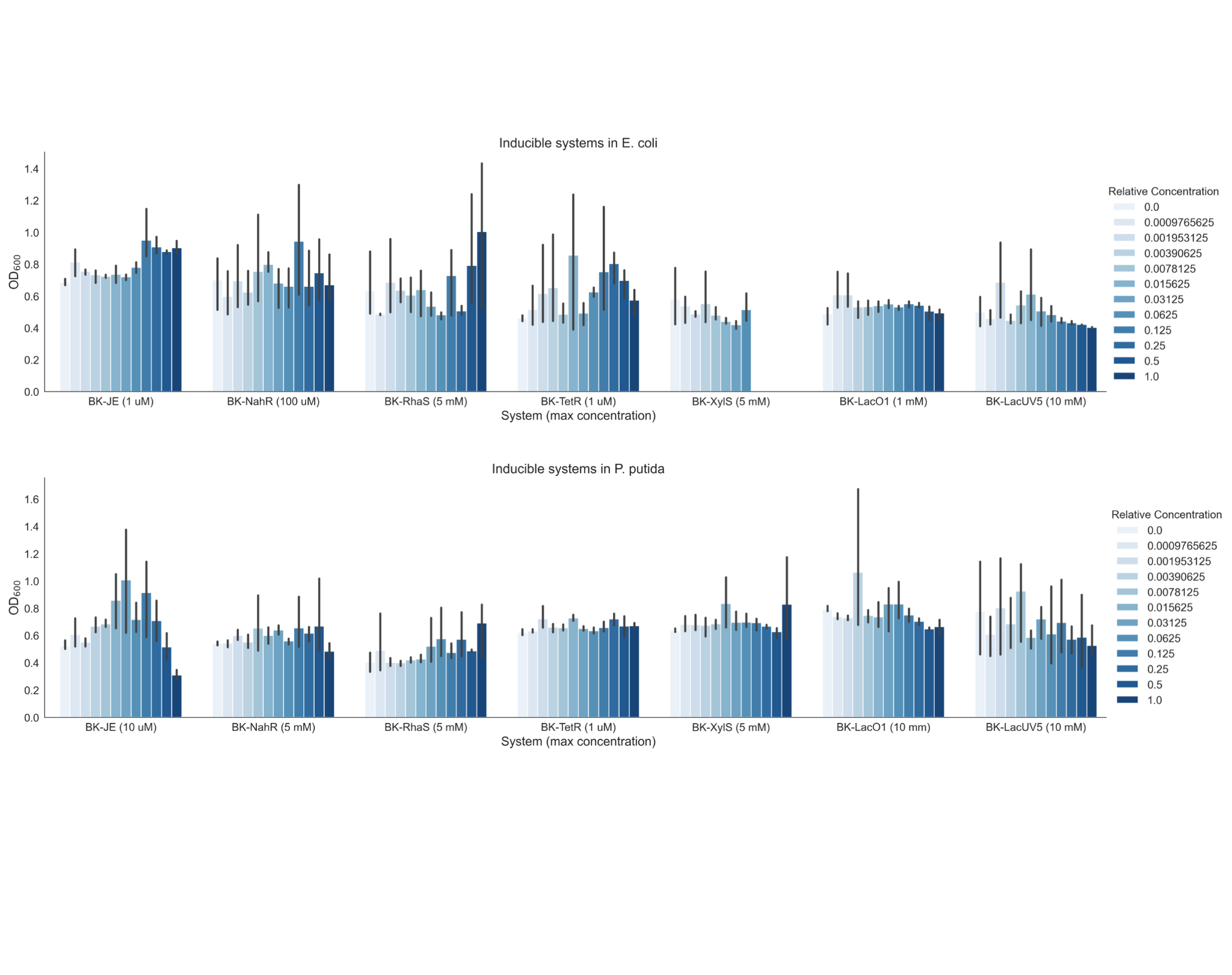
Figure S2:** Corresponding optical density endpoint measurements for data presented in Figure 3 (n=3, error bars = std. dev.)

**
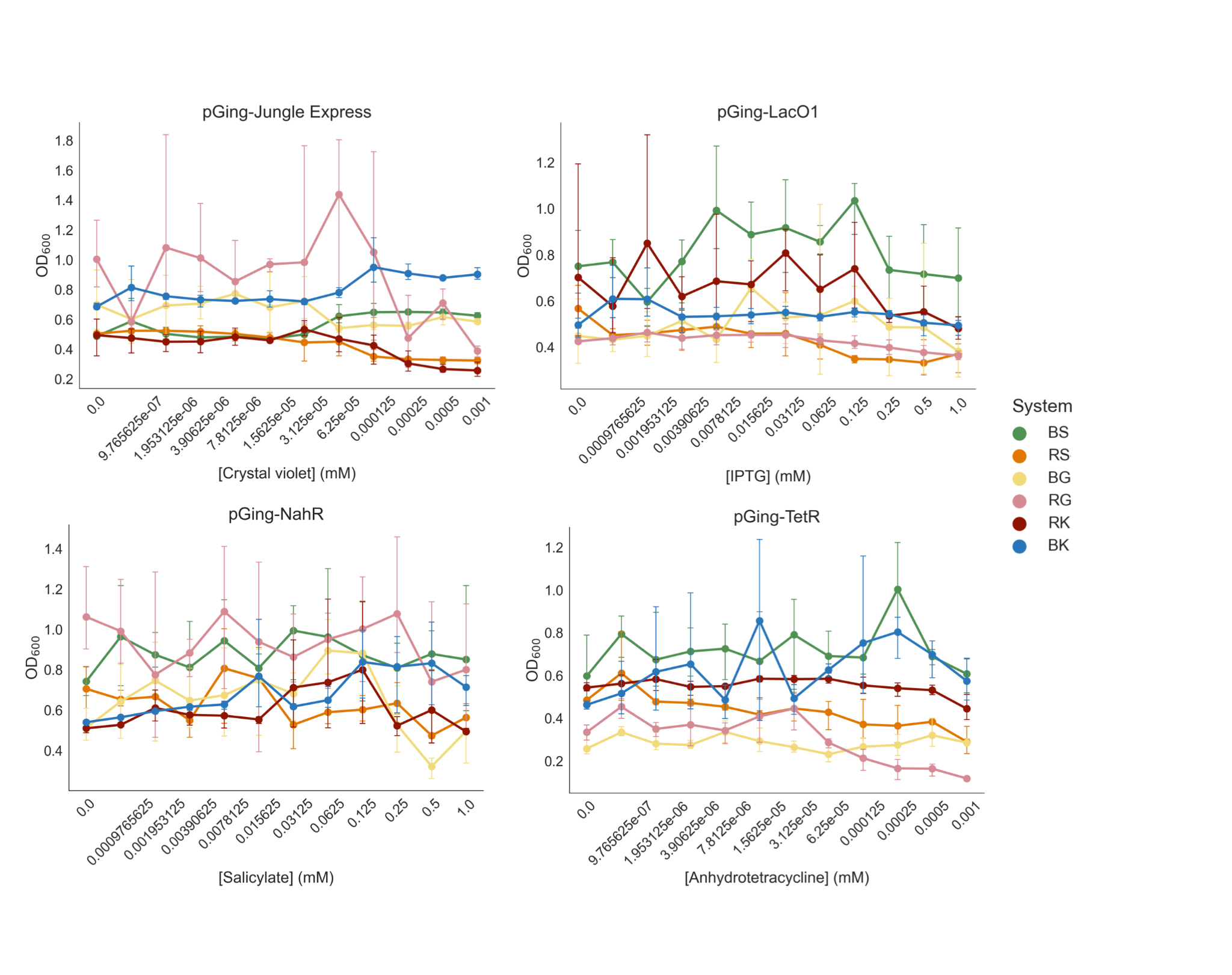
Figure S3:** Corresponding optical density measurements for data presented in Figure 4 (n=3, error bars = std. dev.)


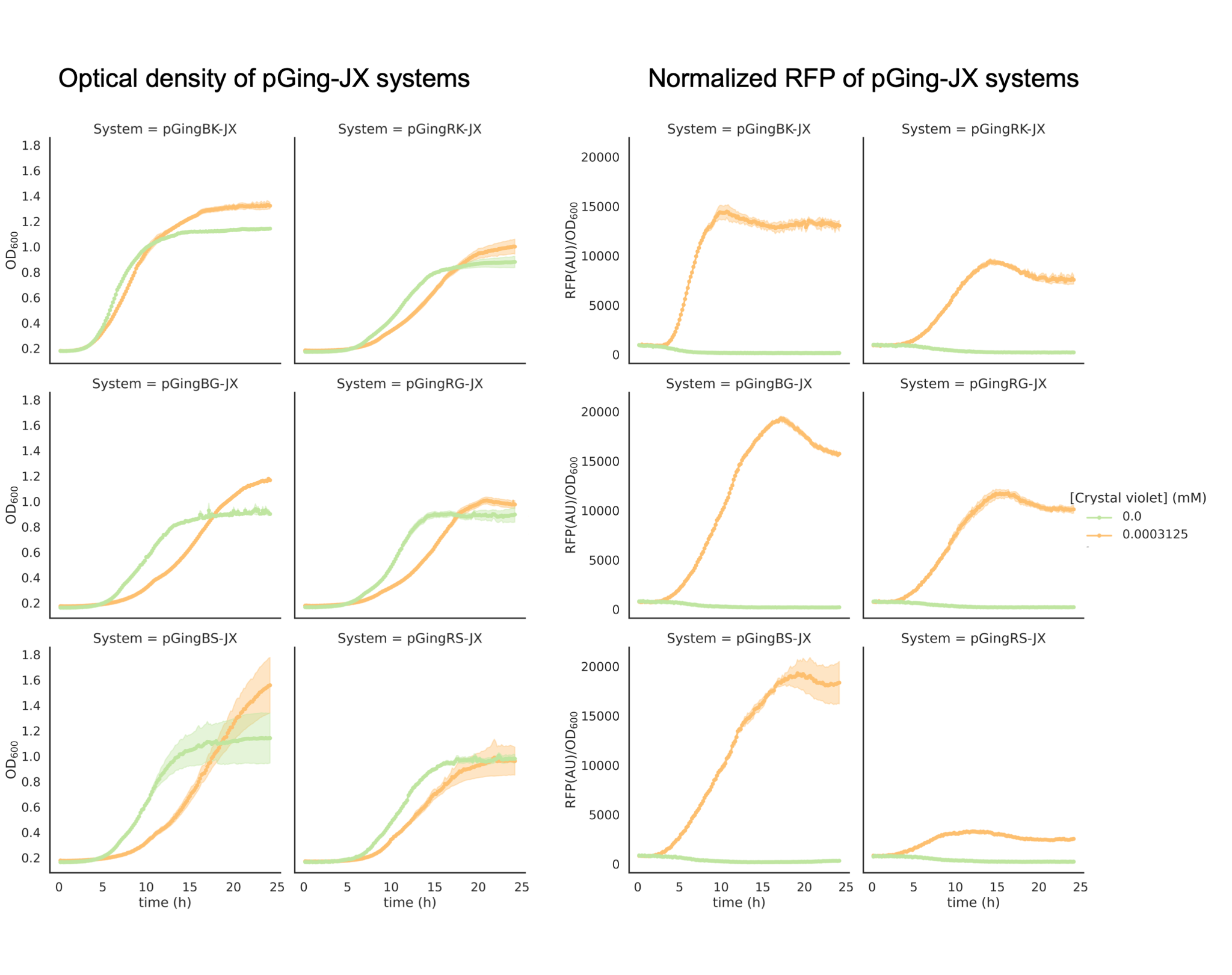
**Figure S4:** Kinetic growth and fluorescence data for pGing-JX systems in *E. coli* (n=3).


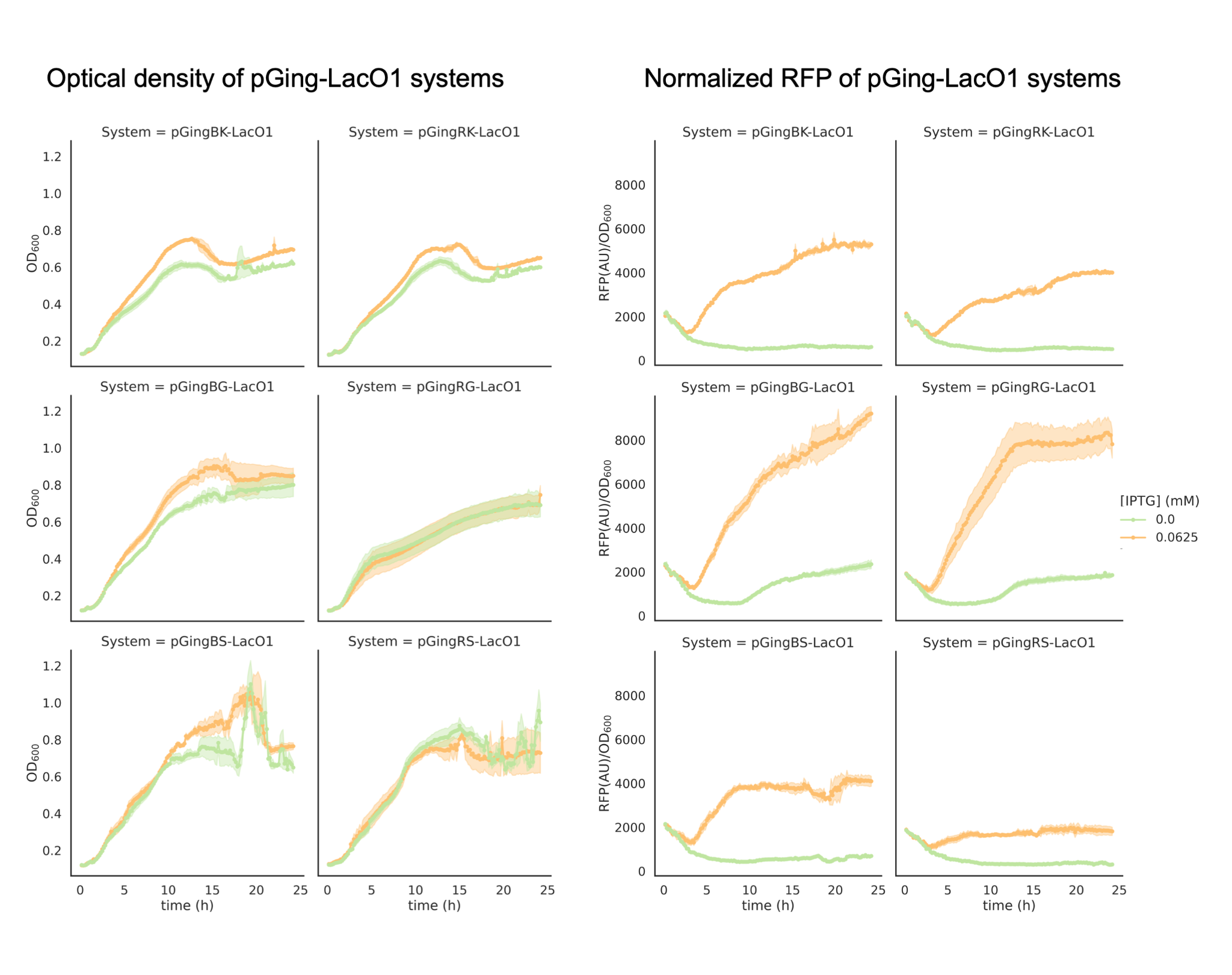
**Figure S5:** Kinetic growth and fluorescence data for pGing-LacO1 systems in *E. coli* (n=3).

**
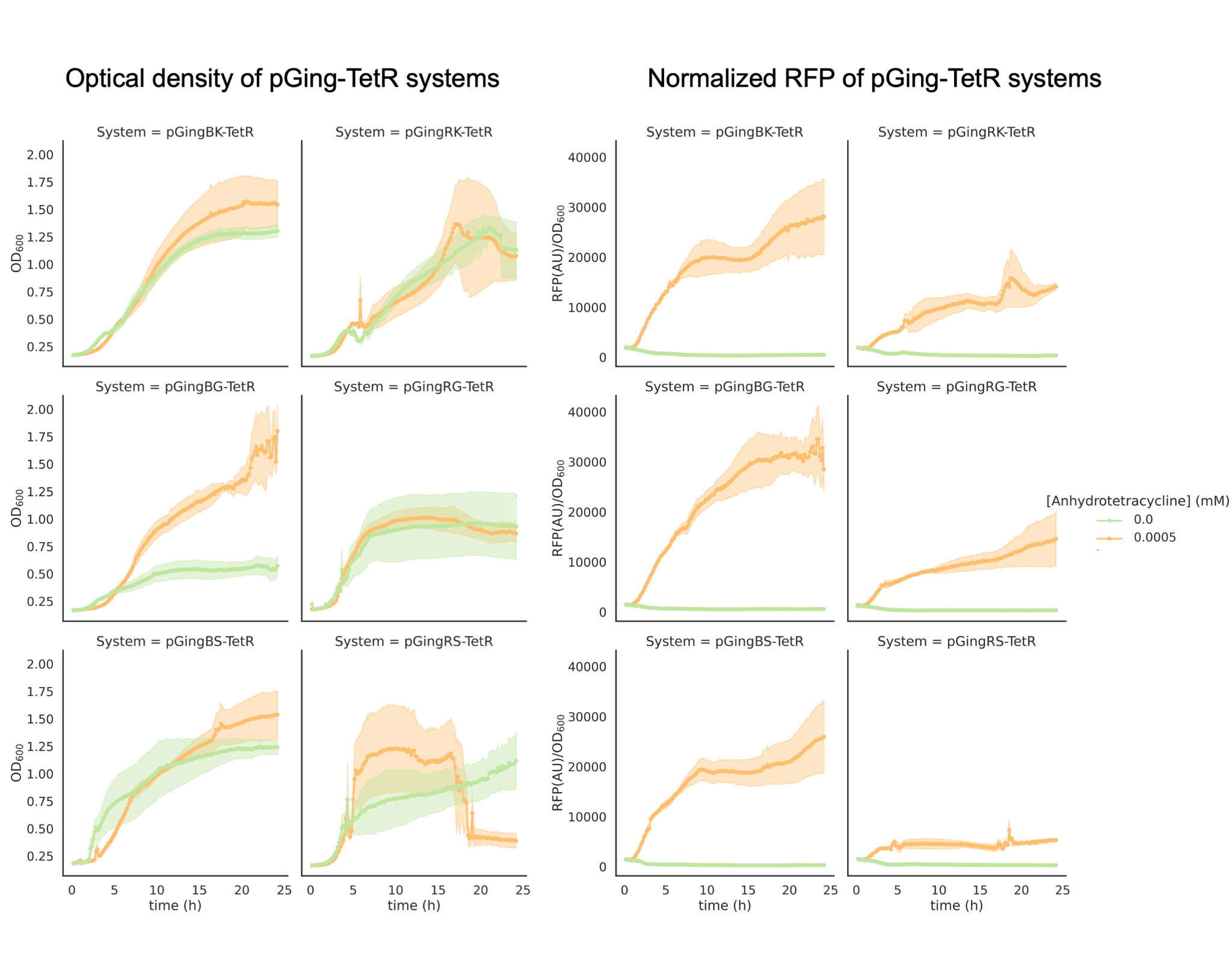
Figure S6:** Kinetic growth and fluorescence data for pGing-TetR systems in *E. coli* (n=3).

**
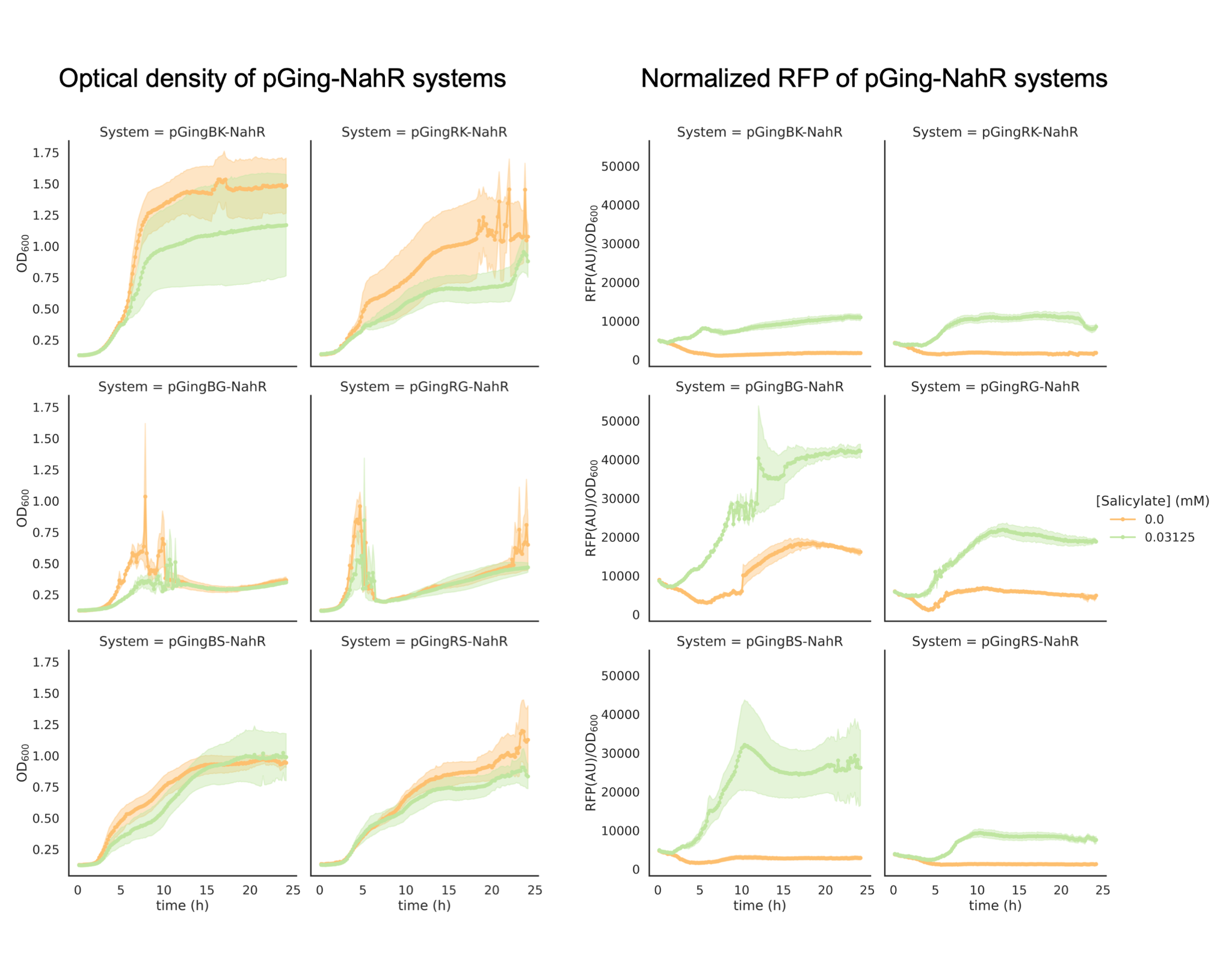
Figure S7:** Kinetic growth and fluorescence data for pGing-NahR systems in *E. coli* (n=3).
